# Early Versus Delayed Laparoscopic Cholecystectomy in Acute Cholecystitis: A Comparative Study

**DOI:** 10.7759/cureus.106111

**Published:** 2026-03-30

**Authors:** Dinesh S., Ashwani Kumar, Dinesh K Pasi, Jaswinder Singh, Parth Dhamija, Naresh Kumar

**Affiliations:** 1 General Surgery, Government Medical College, Patiala, Patiala, IND; 2 Radiodiagnosis, Rajindra Hospital, Government Medical College, Patiala, Patiala, IND

**Keywords:** acute cholecystitis, clinical outcomes, delayed cholecystectomy, early cholecystectomy, laparoscopic surgery, surgical timing

## Abstract

Background: Laparoscopic cholecystectomy is the gold standard for treating symptomatic cholelithiasis. However, the optimal timing of surgery in acute calculous cholecystitis remains a subject of debate. This study aims to compare early versus delayed laparoscopic cholecystectomy to determine the timing that yields better clinical outcomes.

Methods: A prospective comparative study was conducted on 100 patients diagnosed with acute calculous cholecystitis at a tertiary healthcare centre in Punjab, India. Patients were randomized into two groups: Group A underwent early laparoscopic cholecystectomy within 72 hours of symptom onset, while Group B underwent delayed surgery after six to eight weeks of conservative management. Parameters analyzed included demographic data, intraoperative findings, and postoperative outcomes. Statistical analysis was performed to assess significance.

Results: Both groups were comparable in age and gender distribution. Mean operative time did not show a significant difference between groups (p = 0.802). Intraoperative adhesions (24% vs. 18%) and gallbladder rupture (12% vs. 6%) were more frequent in Group A, though not statistically significant. Conversion to open surgery was higher in Group A (8%) compared to Group B (2%), but the difference was not significant (p = 0.359). No biliary tract injuries occurred in either group. Postoperative hospital stay was significantly longer in Group A (3.46 ± 0.54 days) compared to Group B (3.06 ± 0.24 days, p < 0.001).

Conclusions: Early laparoscopic cholecystectomy is a safe and viable option in acute cholecystitis. However, delayed surgery may be associated with marginally lower intraoperative complications and a shorter postoperative hospital stay, suggesting potential benefits in selected cases.

## Introduction

Acute cholecystitis is the inflammation of the gallbladder, typically caused by obstruction of the cystic duct due to gallstones, leading to right upper quadrant pain, fever, and leukocytosis [1]. Laparoscopic cholecystectomy (LC) is the gold standard treatment for symptomatic gallstone disease and acute cholecystitis, offering reduced morbidity and faster recovery compared to open surgery [2]. The timing of surgery remains debated - early LC (within 72 hours) may prevent sequelae of complications and shorten hospital stay, while delayed LC (after six to eight weeks) allows inflammation to subside, potentially reducing operative difficulty [3]. It was considered that early surgery is safe and yields comparable or better outcomes than delayed surgery. Multiple randomized trials and meta-analyses have compared outcomes such as operative time, conversion rates, and complication rates, with varying conclusions. This study aims to compare early and delayed LC in terms of operative time, intraoperative complications, conversion to open surgeries, biliary tract injuries, and postoperative hospital stay.

## Materials and methods

This prospective, randomized comparative study was conducted at a tertiary care teaching hospital between August 2023 and July 2024. Following approval from the Institutional Ethics Committee and in strict adherence to the Declaration of Helsinki, written informed consent was obtained from all participants prior to their enrollment. Gallbladder wall thickness >3 mm and a positive sonographic Murphy’s sign are supportive of acute cholecystitis. CT is useful when ultrasound findings are inconclusive, particularly in obese patients or for detecting complications, while magnetic resonance cholangiopancreatography (MRCP) helps evaluate the biliary tree in suspected choledocholithiasis, and hepatobiliary iminodiacetic acid (HIDA) scan assesses gallbladder function, with non-visualization indicating cholecystitis. Diagnosis is best established by combining clinical and imaging findings. The Tokyo Guidelines provide an evidence-based framework integrating clinical signs, laboratory parameters, and imaging, where diagnosis requires one local sign, one systemic sign, and supportive imaging. Severity is graded into Grade I (mild), suitable for early LC; Grade II (moderate), where early surgery is feasible with appropriate expertise and risk stratification; and Grade III (severe), associated with organ dysfunction, requiring initial supportive management such as antibiotics or percutaneous cholecystostomy followed by delayed surgery. These guidelines enable standardized and severity-based management tailored to institutional capability.

The required sample size was calculated using the standardized formula proposed by Charan and Biswas [4], where n = required sample size, P = estimated prevalence of gallstones in the general population (6.12%, as per Khuroo et al. [5]), q = 100-P = 93.88, d = acceptable margin of error (5%), and Z = 1.96, corresponding to a 95% confidence interval.

Applying the formula: $n = \frac{1.96 \times 1.96 \times 6.12 \times 93.88}{5 \times 5} = 88.29$

Considering an anticipated 10% loss to follow-up and procedural limitations, an additional 10% of the calculated sample (i.e., eight patients) was added. The total sample size was therefore 96. After rounding, the overall sample size was set at 100 patients.

This was a prospective study conducted over a defined period, including all eligible patients presenting with acute cholecystitis between 1 August 2023 and 31 July 2024. To minimize selection bias, two fixed days per week - Tuesday and Friday - were chosen for patient enrollment. After obtaining informed consent, the first qualifying patient for early LC and the first for delayed LC were included sequentially. The study population consisted of 100 adult patients, aged 18 to 70 years, who were diagnosed with acute cholecystitis and scheduled for elective LC. To be eligible, patients had to be fit for general anesthesia and willing to provide consent. Conversely, the study excluded patients with a history of major abdominal surgery, those who were pregnant, patients with known coagulation disorders, or those requiring additional concurrent abdominal procedures.

All surgical procedures followed a standardized four-port laparoscopic technique under general anesthesia. Pneumoperitoneum was established using a Veress needle, followed by the insertion of a 10-mm umbilical camera port. The working ports consisted of a 10-mm epigastric port and two 5-mm ports placed along the midclavicular and anterior axillary lines. During the procedure, the gallbladder was retracted cephalad to facilitate the dissection of Calot’s triangle. Once the cystic artery and duct were identified, clipped, and divided, the gallbladder was dissected from the liver bed and retrieved through the epigastric port. After achieving hemostasis, a 16F suction drain was positioned in the subhepatic space. In our tertiary care center, we handle many difficult LCs; hence, a suction drain is placed routinely as a precaution to detect early bile leak or bleeding, even when no obvious intraoperative complication is noted. The procedure concluded with the removal of ports under direct vision and the release of the pneumoperitoneum.

The primary parameters recorded for comparative analysis included operative time (measured from the initial incision to gallbladder retrieval) and various intraoperative findings such as adhesions, liver bed bleeding, and gallbladder rupture. Additionally, researchers tracked the rate of conversion to open cholecystectomy, postoperative complications, and the total length of hospital stay. Patient recovery was further assessed using Visual Analogue Scale (VAS) pain scores on postoperative days 0 and 1, alongside the monitoring of drain output and the total duration of drain placement. All collected data, including demographics and clinical outcomes, were analyzed using Microsoft Excel 2023 (Microsoft Corp., Redmond, WA, USA). Continuous variables were analyzed and presented as mean values along with their corresponding standard deviations. Categorical data were evaluated using the chi-square test and Fisher's exact test, as appropriate. A p-value of less than 0.05 was considered indicative of statistical significance.

## Results

Patients aged 41-55 years were the most common (32%), with 26% in Group A (early) and 38% in Group B (delayed). Mean ages were 45.02 ± 15.16 years in Group A and 47.26 ± 13.62 years in Group B. The age distribution was statistically non-significant (p = 0.762), indicating well-matched cohorts.

Females predominated both groups: 66% in Group A and 76% in Group B. The gender distribution between groups was statistically insignificant (p = 0.378). However, overall female predominance (71% vs. 29% male) was statistically significant (p = 0.0038) in both groups, reflecting the higher incidence of cholelithiasis among women.

**Figure 1:**
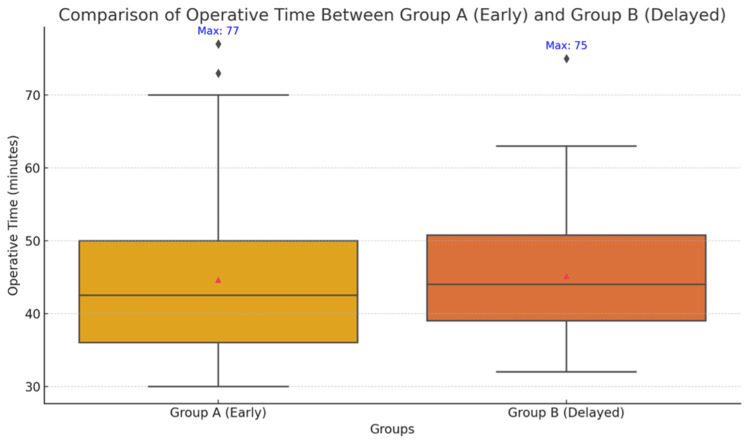
Boxplot graph showing comparison of total operative time (minutes) between the early and delayed groups

Group A had a mean operative time of 44.60 ± 12.22 minutes, and Group B had 45.14 ± 9.07 minutes, with median times of 42.5 and 44 minutes, respectively. Differences were statistically insignificant (p = 0.802), suggesting comparable operative efficiency. The Mann-Whitney U test was used due to non-normal data distribution (Figure 1).

Adhesions were slightly more frequent in Group A (24%) vs. Group B (18%), though statistically non-significant (p = 0.623). Frozen Calot’s triangle occurred more often in early cases (8% vs. 2%), indicating active inflammation. Liver bed bleeding was noted in 16% of Group A and 10% of Group B cases. Most events occurred during Calot’s dissection and gallbladder separation. Differences were statistically insignificant (p = 0.552), suggesting similar intraoperative safety (Table 1).

**Table 1:** Comparison of liver bed bleeding and its occurrence at each stage between early and delayed groups GB: gallbladder

Stage	Early group (A)	Delayed group (B)	p-value
Bleeding during Calot’s dissection	4 (50%)	3 (60%)	0.552
Bleeding during removing GB from the liver bed	3 (37.5%)	1 (20%)	
Bleeding during fundal traction	1 (12.5%)	1 (20%)	
Total bleeding comparison	8 (16%)	5 (10%)	

Gallbladder rupture occurred in 12% of early cases vs. 6% of delayed (p = 1.00). Most ruptures were during extraction. Though higher in Group A, the difference lacked statistical significance (Table 2).

**Table 2:** Comparison of gallbladder (GB) rupture and its occurrence at each stage between early and delayed groups

Stage	Early group (A)	Delayed group (B)	p-value
Rupture during the extraction of the GB	3 (50%)	2 (67%)	1.00
Rupture during fundal traction	1 (16.67%)	0 (0%)	
Rupture during the removal of the GB from the liver bed	1 (16.67%)	1 (33%)	
Rupture during Calot’s dissection	1 (16.67%)	0 (0%)	
Total rupture comparison	6 (12%)	3 (6%)	

Stone spillage was slightly more in the early group (6%) vs. the delayed (4%), with no significant difference (p = 1.00). Extraction was the most common stage for spillage (Table 3).

**Table 3:** Comparison of stone spillage and its occurrence at each stage between early and delayed groups GB: gallbladder

Stage	Early group (A)	Delayed group (B)	p-value
Stone spillage during the extraction of the GB	2 (67%)	1 (50%)	1.00
Stone spillage during the removal of the GB from the liver bed	0 (0%)	0 (50%)	
Stone spillage during Calot’s dissection	1 (33%)	1 (50%)	
Total stone spillage comparison	3 (6%)	2 (4%)	

No biliary tract injuries occurred in either group, which may be due to the adoption of safe surgical techniques by the team. Conversion to open surgery was required in four cases in Group A and one in Group B. Though higher in the early group (8% vs. 2%), the difference was not statistically significant.

**Figure 2:**
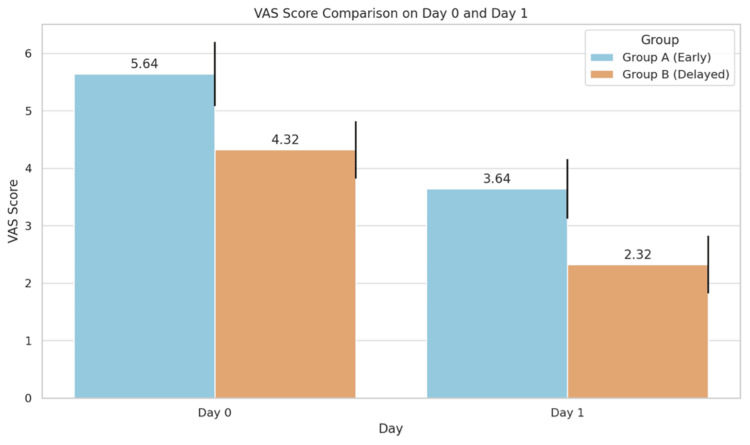
Bar graph showing comparison of VAS scores on POD 0 and POD 1 between early and delayed group VAS: Visual Analog Scale; POD: postoperative day

Postoperative pain was higher in Group A. On day 0, mean VAS was 5.64 ± 0.56 in Group A vs. 4.32 ± 0.47 in Group B (p < 0.0001). On day 1, VAS was 3.64 ± 0.56 vs. 2.32 ± 0.47, respectively (p < 0.0001), indicating statistically significantly higher pain in the early group (Figure 2).

**Figure 3:**
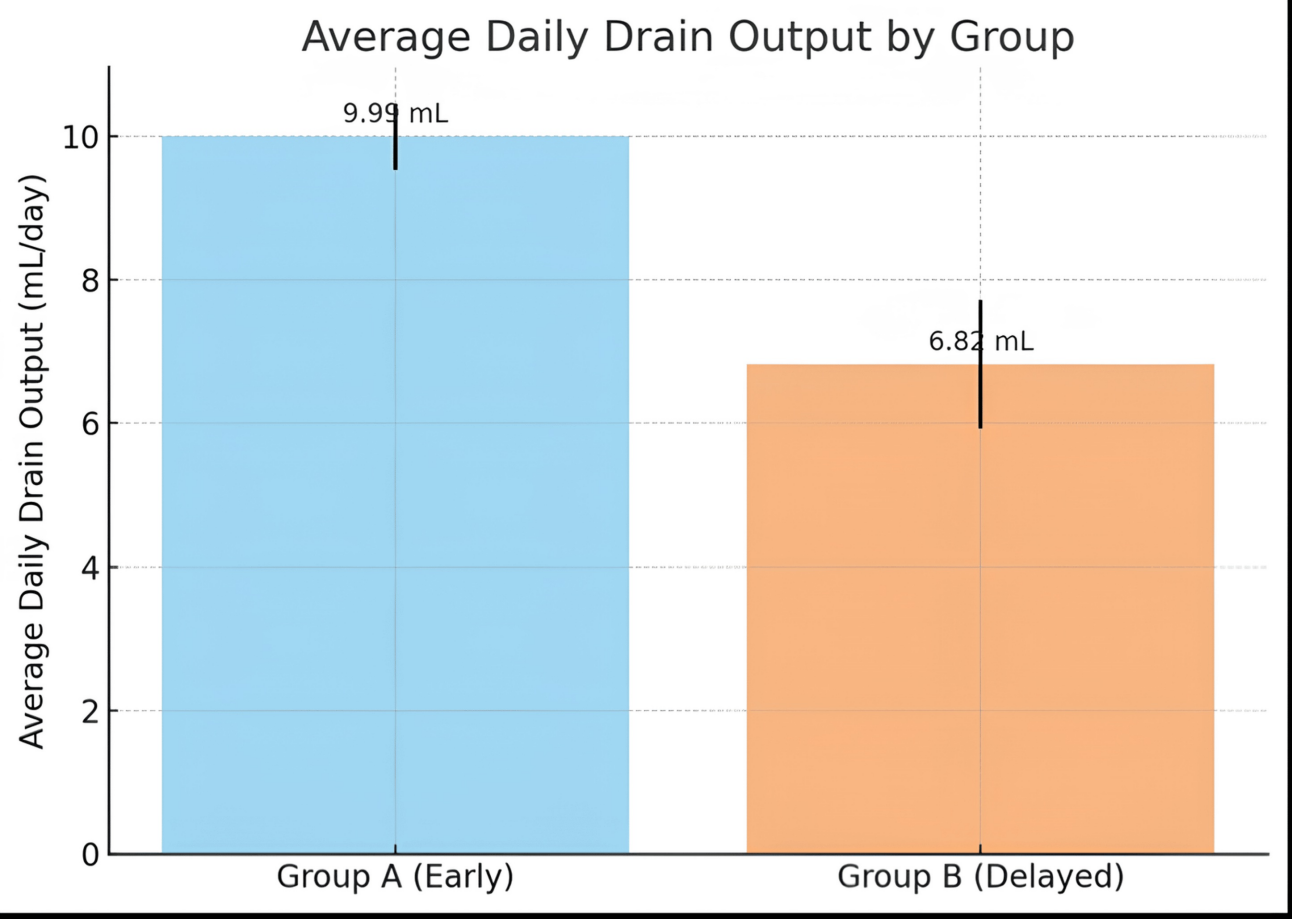
Bar graph comparing average daily drain output between early and delayed groups

Average daily drain output was significantly higher in the early group (9.99 ± 0.46 mL) vs. the delayed group (6.82 ± 0.89 mL) with p < 0.0001. This may be attributed to inflammation and tissue trauma in early cholecystectomy (Table 4 and Figure 3).

**Table 4:** Comparison of average daily drain output between early and delayed groups

Group	Mean	Median	Min	Max	p-value
Group A (early)	9.99 ± 0.46	10.00	7.5	12.0	<0.0001
Group B (delayed)	6.82 ± 0.89	6.67	5.0	10.0	<0.0001

**Figure 4:**
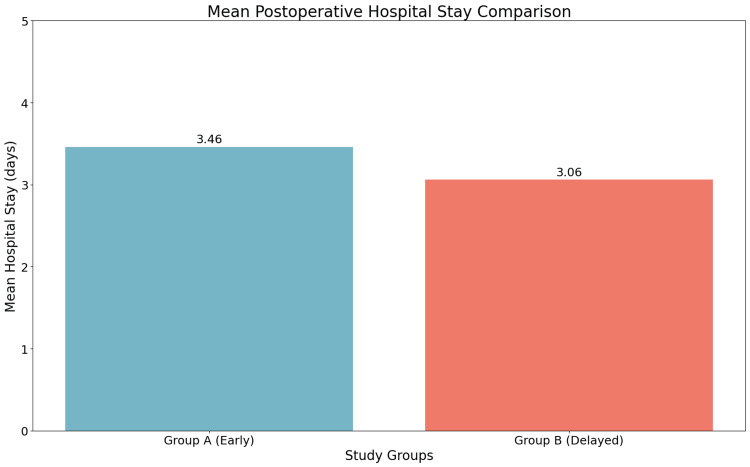
Comparison of postoperative hospital stay duration between early and delayed groups

Group A had a longer mean postoperative hospital stay (3.46 ± 0.54 days) than Group B (3.06 ± 0.24 days), despite a median stay of three days in both. This difference was highly significant (p = 0.000006), suggesting that early cholecystectomy is associated with a longer recovery period, likely due to increased postoperative drainage and complications (Table 5 and Figure 4).

**Table 5:** Comparison of postoperative hospital stay duration between early and delayed groups

Group	Mean ± SD	Median	IQR	Min	Max	p-value
Group A (early)	3.46 ± 0.54	3.0	3.0-4.0 (IQR: 1.0)	3	5	0.000006
Group B (delayed)	3.06 ± 0.24	3.0	3.0-3.0 (IQR: 0.0)	3	4	0.000006

## Discussion

This prospective comparative study was conducted at a tertiary healthcare centre in Punjab, India, to evaluate and compare outcomes of early versus delayed LC in acute cholecystitis. Fifty patients were enrolled in each group, and multiple perioperative parameters were assessed and analyzed.

Operative time

Mean operative time was 44.60 ± 12.22 minutes in Group A and 45.14 ± 9.07 minutes in Group B (p = 0.802), indicating no significant difference. This aligns with studies by Mudhale et al. (2023) [6] and Budisca et al. (2024) [7], while studies like Prasad et al. (2023) [8] demonstrated significantly shorter operative times for early intervention. Operative times in our study were notably shorter overall, which may reflect institutional surgical expertise and fewer complications.

Intraoperative adhesions

Adhesions were more frequent in the early group (24%) compared to the delayed group (18%), though not statistically significant (p = 0.623). These findings are consistent with Anwar et al. (2022) [9] and Chand et al. (2024) [10], though Shah et al. (2020) [11] reported a higher incidence in delayed surgeries. Frozen Calot’s triangle was notably more common in the early group, possibly due to active inflammation.

Liver bed bleeding

Liver bed bleeding occurred in 16% of Group A and 10% of Group B (p = 0.623). This finding aligns with Anwar et al. (2022) [9] and Chand et al. (2024) [10], supporting that early surgery may pose a higher bleeding risk due to inflamed tissue. However, Shah et al. (2020) [11] reported more bleeding in delayed cases. Effective hemostatic measures in both groups ensured safe outcomes.

Gallbladder rupture

Gallbladder rupture was more frequent in the early group (12%) compared to the delayed group (6%), but the difference was not significant (p = 1.00). Similar findings were seen in Rather et al. (2020) [12] and Chand et al. (2024) [10]. Sivakumar et al. (2023) [13], however, reported higher rupture rates in delayed surgeries. Increased rupture in the early group may be attributed to friability during acute inflammation.

Conversion to open surgery

The early group had a conversion rate of 8% versus 2% in the delayed group (p = 0.359), consistent with Thomas et al. (2023) [14] and Sharma et al. (2020) [15]. Nasir et al. (2021) [16], however, observed more conversions in delayed surgeries, suggesting case selection and surgical expertise influence outcomes more than timing alone.

Biliary tract injuries

No biliary tract injuries occurred in either group, which may be due to the adoption of safe surgical techniques to be used during laparoscopic surgeries as per routine protocol. This corresponds with Sivakumar et al. (2023) [13] and reflects surgical expertise and early decision for conversion when needed. Studies by Shah et al. (2020) [11] and Zainai et al. (2023) [17] reported minor injuries, especially in early cases, highlighting the importance of technique over timing.

Postoperative hospital stay

Mean hospital stay was significantly longer in the early group (3.46 ± 0.54 days) compared to the delayed group (3.06 ± 0.24 days), with a highly significant p-value (<0.001). This contrasts with most studies, such as Thomas et al. (2023) [14] and Sukhontamas (2020) [18], which found reduced stay in early surgery. In our study, prolonged drainage in early cases might explain the extended hospital stay.

## Conclusions

This prospective comparative study concludes that both early and delayed LC for acute cholecystitis offer comparable intraoperative safety, with no significant differences in conversion rates or biliary complications. While early intervention is technically feasible and safe, it was associated with higher postoperative drain output, increased pain scores, and longer hospital stays, likely due to the acute inflammatory milieu. Ultimately, both approaches are valid strategies; however, surgical timing should be individualized based on the patient's inflammatory severity and institutional logistics to optimize postoperative recovery.
